# Supplementary material for: Exposing new taxonomic variation with inflammation — a murine model-specific genome database for gut microbiome researchers
Source: Microbiome. 2023 May 20;11:114. doi: 10.1186/s40168-023-01529-7 (PMC10199544; doi:10.1186/s40168-023-01529-7)
Supplement: Supplementary file 9 — Additional file 8: Fig. S1. Relative abundance of classes in pre-infection communities are not statistically different than uninfected communities, indicating a shared starting microbiome prior to infection. Fig. S2. CBAJ-DB uninfected (no Salmonella) amplicon sequenced communities show considerable taxonomic overlap with communities from other CBA studies. Fig. S3. A) Gigabase pairs (Gbps) per sample of prevalent murine genome databases. Fig. S4. Contamination and completion statistics and the most resolved taxonomy groups for MAGs containing amplicon sequencing variants (ASVs). Fig. S5. Procrustes analysis of dereplicated medium and high quality (dMQHQ) metagenome assembled genomes (MAGs) and viral metagenome assembled genomes (vMAGs). [file 40168_2023_1529_MOESM8_ESM.docx]

***Supplementary Information for “Exposing New Taxonomic Variation with Inflammation – A Murine Model-Specific Genome Database for Gut Microbiome Researchers“***

*Ikaia Leleiwi^1,2^; Josue Rodriguez-Ramos^2,3^; Michael Shaffer^2^; Anice Sabag-Daigle^4^; Katherine Kokkinias^2,5^; Rory M Flynn^2^; Rebecca A Daly^2^; Linnea FM Kop^6^; Lindsey M Solden^2^; Brian M. M. Ahmer^4^; Mikayla A Borton^2^; Kelly C Wrighton^1,2,3,5*^*

*Authors and Affiliations*

*^1^ Department of Cell and Molecular Biology, The Colorado State University, Fort Collins, CO, USA*

*^2^ Department of Soil and Crop Sciences, The Colorado State University, Fort Collins, CO, USA*

*^3^ Graduate Degree Program in Ecology, The Colorado State University, Fort Collins, CO, USA*

*^4^ Department of Microbial Infection and Immunity, The Ohio State University, Columbus, OH, USA*

*^5^ Department of Microbiology, Immunology, and Pathology, The Colorado State University, Fort Collins, CO, USA*

***Supplementary Figures***

Fig S1


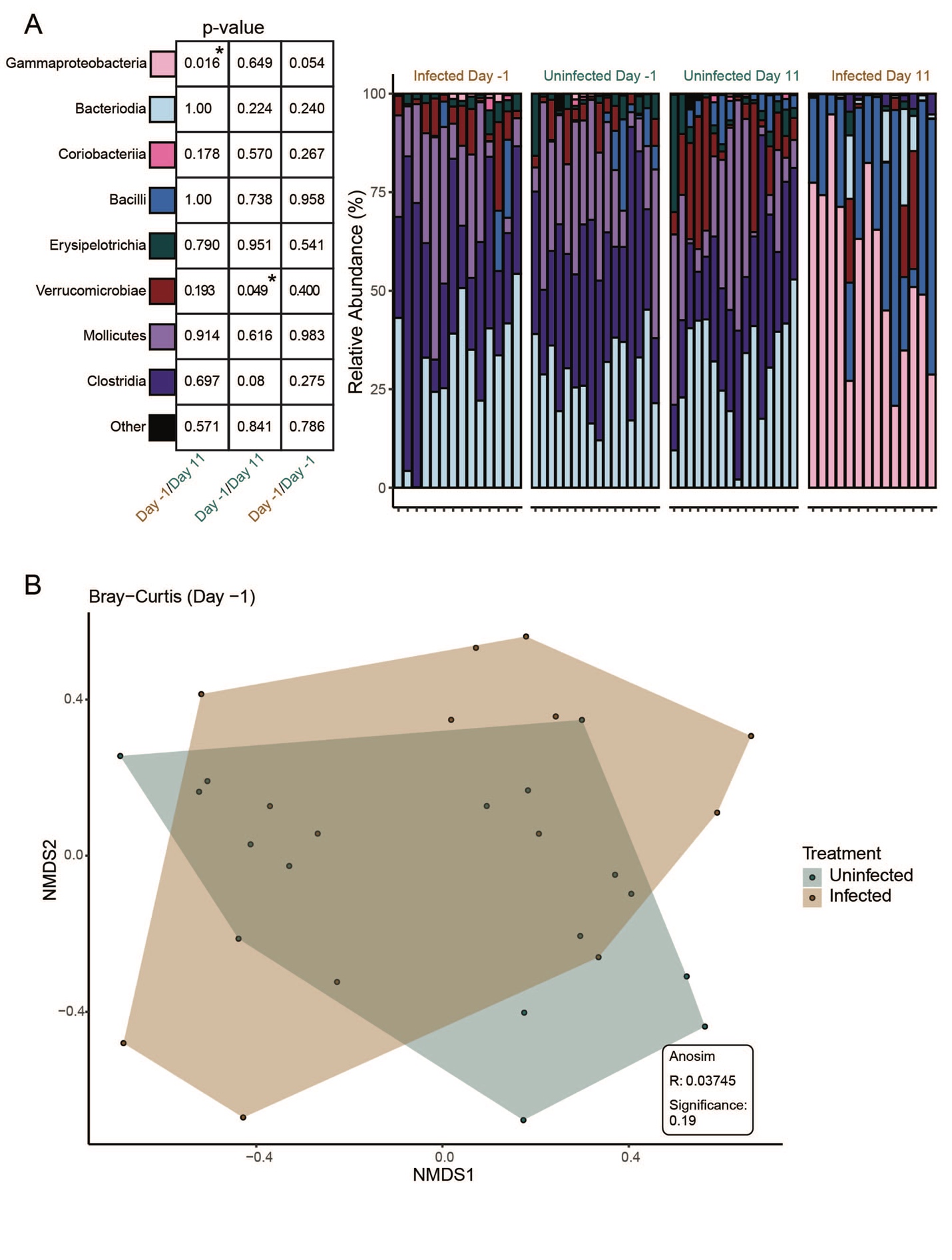


**Fig. S1 Relative abundance of classes in pre-infection communities are not statistically different than uninfected communities, indicating a shared starting microbiome prior to infection.**

A) Wilcoxon Rank Sum significance revealing no difference between Infected Day -1 communities and uninfected communities from either Day -1 or Day 11. Summed abundance within each class was examined comparing different timepoints and treatments (uninfected = green, infected = brown). B) NMDS of Day -1 communities colored by treatment. No significant difference exists between communities of mice from either treatment at Day -1 as determined by Anosim.

Fig. S2


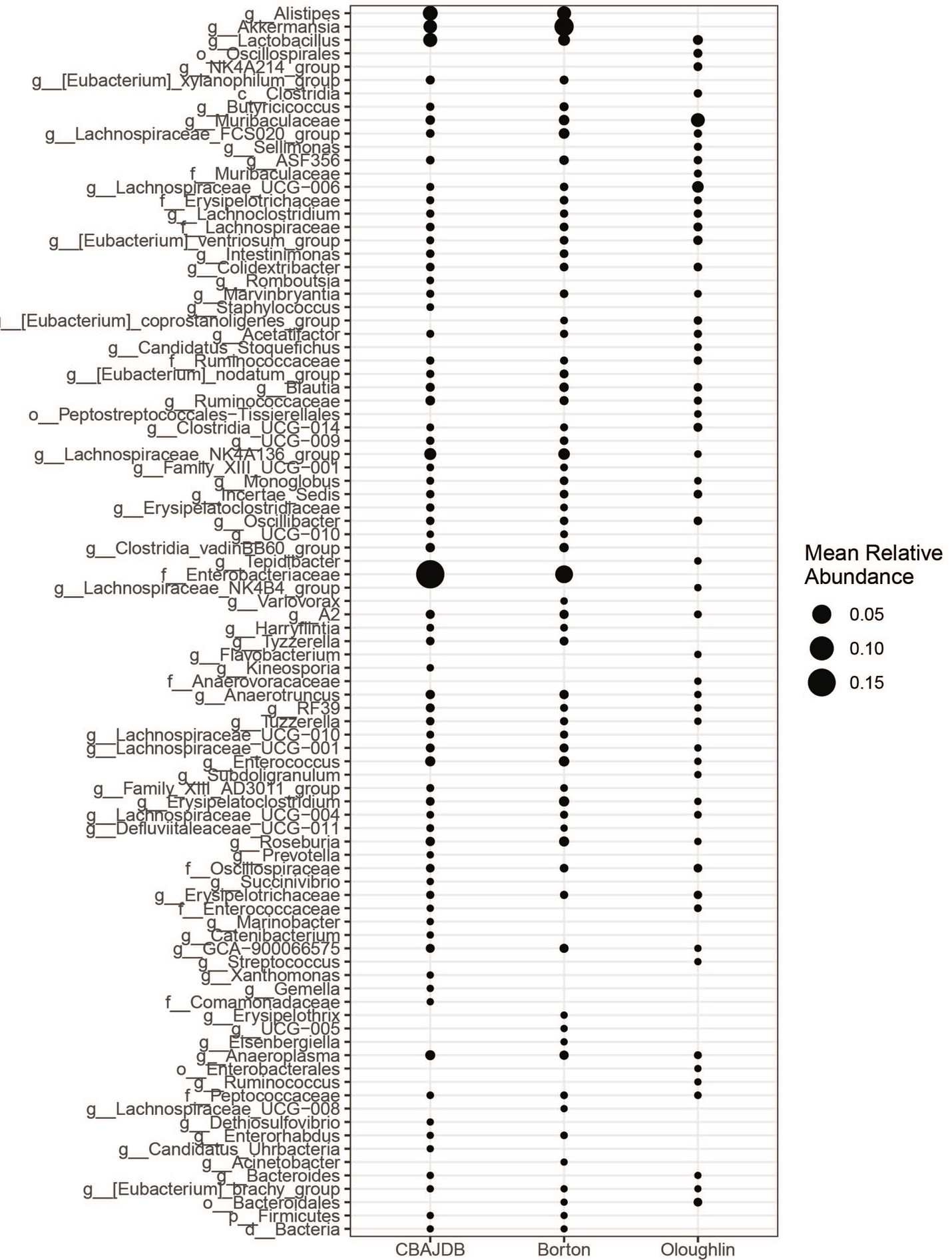


**FigS2. CBAJ-DB uninfected (no *Salmonella*) amplicon sequenced communities show considerable taxonomic overlap with communities from other CBA studies.** Points indicate lowest meaningful taxonomy presence in CBA mice from the CBAJ-DB, Borton et.al. 2017 [27], or O’loughlin et.al. 2015 [28]. Points are sized by average relative abundance of ASVs contributing to each taxonomy.

Fig. S3


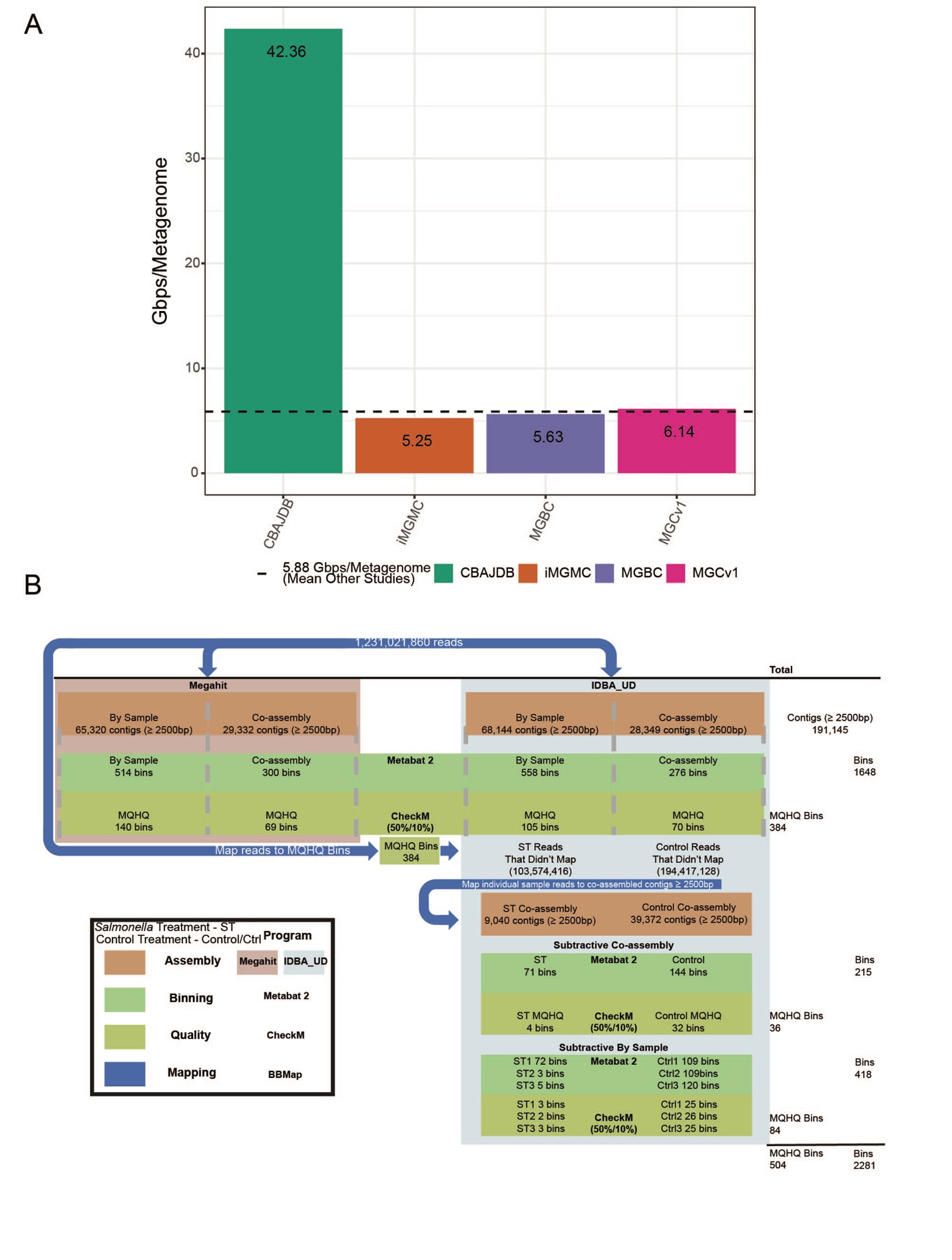


**FigS3. A) Gigabase pairs (Gbps) per sample of prevalent murine genome databases**. integrated Mouse Gut Metagenomic Catalog (iMGMC), The Mouse Gastrointestinal Bacterial Catalogue (MGBC), and Xiao et.al. 2015 (<https://doi.org/10.1038/nbt.3353>) (MGCv1) compared to the per sample sequencing effort of the CBAJ-DB. **B) Assembly and binning workflow.** Total and medium and high quality (MQHQ) bins derived from Megahit assembler (left) and IDBA_UD assembler (right) shown, blue arrows indicate read mapping for subtractive assembly.

Fig. S4


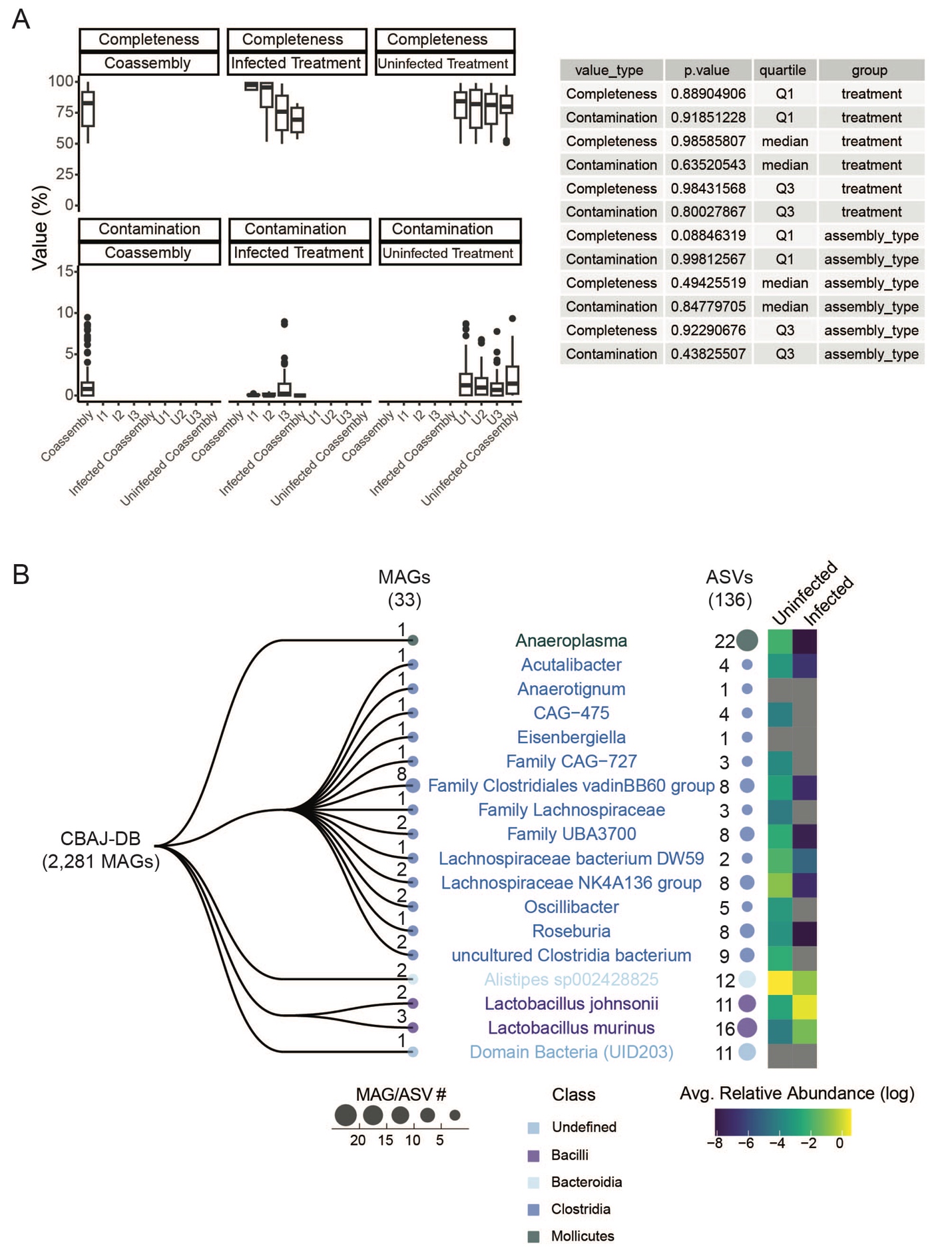


**Fig. S4 Contamination and completion statistics and the most resolved taxonomy groups for MAGs containing amplicon sequencing variants (ASVs).** A) Distribution of contamination and completion of medium and high quality MAGs by treatment and assembly method. Table shows Chi Squared test p-values of quartiles between groups. B) MAG groups with matching ASVs from 16S rRNA sequencing as determined by Mmseqs2 and Barrnap (see methods). Colored text indicates the lowest resolved taxonomy. Mean ASV relative abundance within each taxonomy group in each treatment is represented in Log relative abundance (right).

Fig. S5


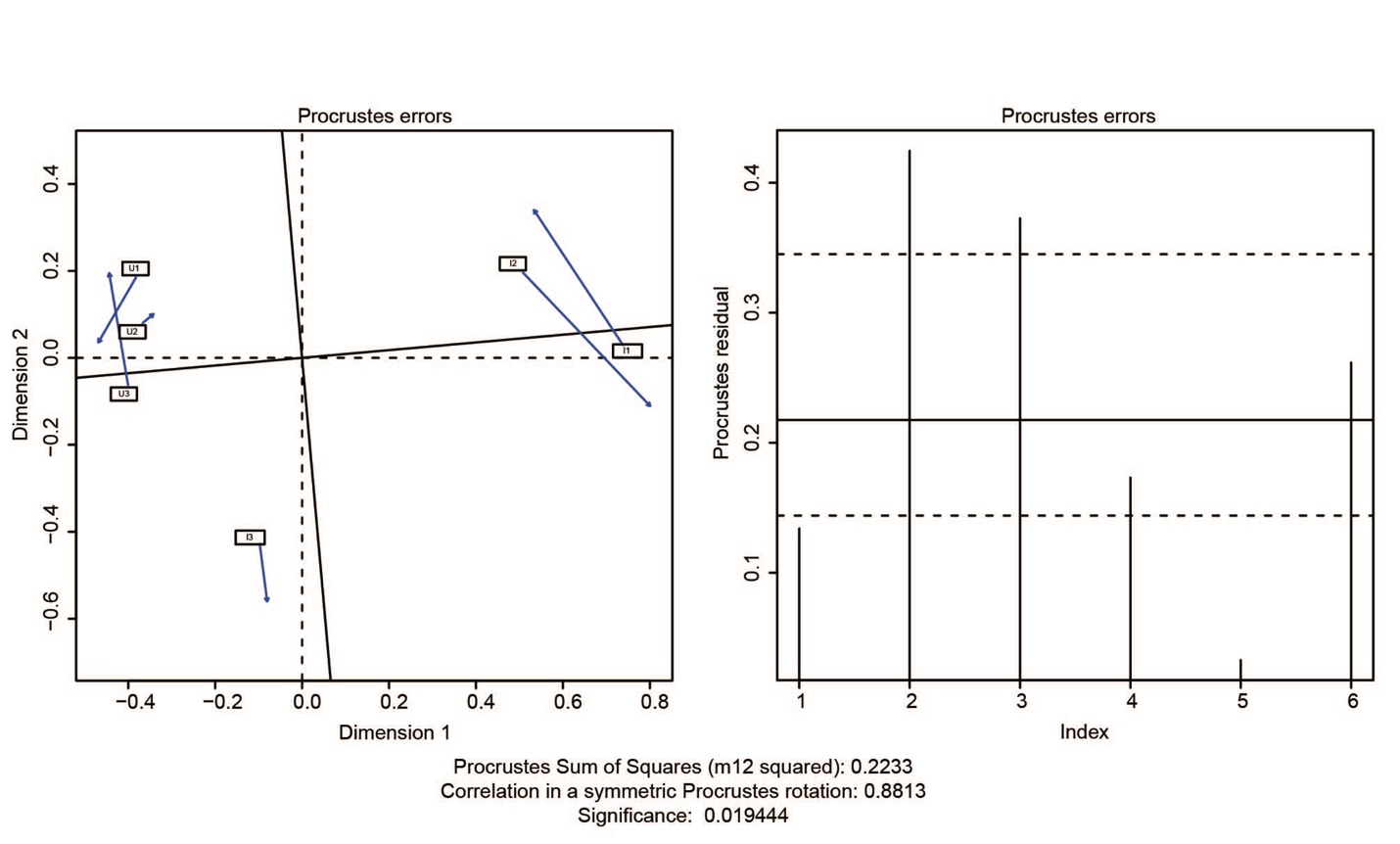


**Fig. S5 Procrustes analysis of dereplicated medium and high quality (dMQHQ) metagenome assembled genomes (MAGs) and viral metagenome assembled genomes (vMAGs)**. dMQHQ and vMAG relative abundance non-metric multidimensional scaling (NMDS) and viral genome-database NMDS showing high significant similarity of ordinations.

***Additional File Descriptions***

Additional file 1: Data S1, S1_16S_Data_SOM.xlsx

Additional data pertaining to 16S rRNA amplicon sequencing including IDs of mice sequenced and their treatment (sheet: 16S_rRNA_Metadata). The file also includes sheets with Lipocalin-2 measurements (sheet: Lipocalin_2), ASV abundances (sheet: ASV_table), ASV taxonomy (sheet: Taxonomy (Silva 132)), and 16s sequences found in MAGs (sheet: 16S_in_MAGs).

Additional file 2: Data S2, S2_CBAJDB_SOM.xlsx

MAG IDs of medium and high-quality MAGs along with their completeness and contamination scores, bin size, and contig information (sheet: MQHQ_stats). tRNA location in each bin (sheet: MQHQ_tRNA) and sampling depth (sheet: Sampling_Depth) along with read mapping results (sheets: Mapping (95%), Mapping (strict)).

Additional file 3: Data S3, S3_MAG_annotations_MQHQ.tsv

DRAM gene annotations of the MQHQ MAG in the CBAJ-DB.

Additional file 4: Data S4, S4_vMAG_master_spreadsheet_v8.xlsx

All data pertaining to the recovered vMAGs in the CBAJ-DB including the dereplicated vMAG set (sheet: UViG_info_final_609), read mapping abundances (sheet: read_mapping_abunces_609_vMAGs), host linkages (sheet: virus_host_linkages) and AMG information (sheet: 36_host_linked_AMGs).

Additional file 5: Data S5, S5_OTHER_DB_SOM.xlsx

dRep and FastANI clustering results of CBAJ-DB with iMGMC, MGBC, and human MAGs.

Additional file 6: Data S6, S6_HUMAN_READS_SOM.xlsx

Read mapping data from Jason Lloyd Price cohort and human cohort mapped to the CBAJ-DB.

Additional file 7: Data S7, S7_FUNCTIONS_RULES_SOM.xlsx

Individual MAG function presence and relevant gene counts along with the rule sets used to assign genome functionality.
